# Clerical Quackery

**Published:** 1859-12

**Authors:** O. C. Gibbs

**Affiliations:** Frewsburgh, N. Y.


### ART. IV.—*Clerical Quackery*, by O. C. Gibbs, M. D., Frewsburgh, N. Y.

There are, perhaps, no persons who exercise more influence in their respective communities than the clergy. The ignorant and the vulgar particularly, look up to their minister for light and correct opinions upon all matters, whether concerning spiritual or temporal things. There are thousands and tens of thousands in our boasted land of liberty and enlightenment, who are in the most abject mental servitude—wearing their priestly chains with great complacency, voting as their pastor tells them is right—and trust their lives in the hands, and gild the pockets, of such quacks who happen to be fashionable at the time with him who meekly dispenses religious ceremonies. Why ministers of the gospel of Christ, who should be members of an honest, liberal, high-minded, and learned profession, should see it their duty to eschew medical literature, science, and learning, and give their influence to the support of ignorance, quackery, and the most arrogant pretenders, is beyond human comprehension. There are, scattered over the land, many honorable exceptions, who give to the educated, high-minded, and honorable physician, who has devoted his life, and all his mental and physical energies to the welfare of the race, the right hand of fellowship and encouragement.

The above thoughts were suggested on listening to a public discourse by a talented divine, in which he gave expression to the following idea: "Physicians are a useless excrescence, an expensive vampire, upon society. When a man's time has come, he will die in spite of all the physicians in the world ; and when his time has not come, however sick he may be, he will recover as well without, as with, medical aid." Against such doctrines we enter our decided protest, as they develop the most objectional feature of fatalism.

Why does not their advocate act honestly, discontinue his ministerial labors, and relinquish his comfortable salary? It is a legitimate deduction from the doctrine to say, that those God has designed to save will be saved without preaching, and those whom he has not designed to save, no amount of ministerial labor can affect. If his child should fall from a bridge into the middle of a river, would he stand composedly upon the shore and reason thus: "If his time has come he will drown, in spite of all my efforts to save; and if it has not come, he will get out some way, without my wetting myself to accomplish his rescue." The parent who would reason thus, would be considered a brute, or a fit subject for a mad-house. The clergyman that will use his ministerial influence, in the sacred pulpit and in private intercourse, to the disparagement of medical science,—who will say that the administration of medicine is always worse than useless, and that physicians are an expensive excrescence upon society, we consider either a knave or a fool.

There are thousands of deaths annually which are insultingly ascribed to the providence of God, that are really attributable to the foolishness of man. Christ, our great exsampler, used instrumentalities for the cure of the afflicted: the eyes of the blind were anointed, and the leprous were sent to wash repeatedly in the pool of Siloam.

There is no class of men who receive so many gratuities at the hands of physicians, as the members of the clerical profession; and, according to our observation, there is no equal number of intelligent men that so abuse their influence to the injury of regular medicine.

In our little village, we support four clergymen. One of them gave expression to the quotation given above; one is a Thompsonian, one a Homœopathist, and one a believer in the all-healing virtues of cold water. Neither has any scruples to use his ministerial influence for the advocacy of his own peculiar doctrines.

In our first three years of medical practice, we were followed to the bedside of almost every sick one, by a clergymen, who had no compunctions of conscience in countermanding our orders, and advising the various uses of water instead. On one occasion, he called on a good sister, and greatly to his surprise, found her laboring under great pain in her bowels. He advised her not to delay another day, but to go to the Water Cure (some 60 miles distant), where a perfect and permanent cure would soon be effected. She said she had sent for her physician, in whom she had confidence, and had no doubt she should soon be better without the necessity of leaving home. He urged the matter still, and with such warmth and pertinacity, that one of the good mothers was obliged to inform him that the lady was in the first stage of labor, and that her physician would soon be there, and that his visit would be more acceptable at some other time. Two years later, the clergyman's lady attempted to superintend the accouchment of this same woman; and death to both mother and child was the result.

In either of the professions, the field of labor and of study is wide enough for the ambition of any man, however extended his researches or capacious his powers. Having a common origin and a common object—originating in sin, and having for their object the amelioration of the physical, moral, and social conditions of the race—there should exist in the professional *trio*, a bond of union and of sympathy, surpassed nowhere else in science or in social relations. Man can, if he will, deal justly with his fellows, in which event, if universal, the profession of law would be a superfluity; with the Divine Law and God's revealed will before him, he might so attune his actions, in conformity with the healthy existence of the soul, as not to require the services of doctors of divinity; but say what we will, the profession of medicine is a necessity that cannot well be dispensed with. Man's nature gravitates towards the grave, and disease and pain are casualties by the way, depending upon causes ever at work, over which the human judgment has, at least, but limited control. Cuts and bruises, dislocated joints and broken bones, no human foresight can wholly prevent. The poisoned air of an unhealthy climate, and the vicissitudes of an unpropitious season, are beyond human agency and control. The breeze that brings upon its wings the seeds of the pestilence and the plague, can only be rendered salubrious, if at all, by such instrumentalities as the science of medicine has only made known. "Then give place to the physician, for the Lord hath created him: let him not go from thee, for thou hast *need* of him," said one of old; and that *need* has and will continue, so long as children are born, whose very cradles rock diseaseward, and whose every subsequent footstep is toward the grave.

Our clerical friends should remember that physicians have had no light of revelation to guide them, but have been obliged to search for truth in the wilderness of ignorance; their very way hedged in by prejudice, superstition, legal enactments, and penal enjoinders. If they see us giving countenance to what they suppose to be errors, let them check their vanity, by inquiring how long it is since women were hung or burned for witchcraft by *their* sanction.

